# Prognostic Value of Perioperative Near-Infrared Spectroscopy Monitoring for Postoperative Acute Kidney Injury in Pediatric Cardiac Surgery: A Systematic Review

**DOI:** 10.1177/10892532251316682

**Published:** 2025-02-10

**Authors:** Cornelia K. Niezen, Marco Modestini, Dario Massari, Arend F. Bos, Thomas W. L. Scheeren, Michel M. R. F. Struys, Jaap Jan Vos

**Affiliations:** 1 Department of Anesthesiology, 10173 University Medical Center Groningen, University of Groningen, Groningen, The Netherlands; 2 Department of Anesthesiology, Isala Hospital, Zwolle, The Netherlands; 3 Department of Neonatology, Beatrix Children’s Hospital, 10173 University Medical Center Groningen, University of Groningen, Groningen, The Netherlands; 4 Department of Basic and Applied Medical Sciences, Ghent University, Gent; Belgium

**Keywords:** NIRS, AKI, cardiac anesthesia, pediatric intensive care, cardiac surgery, cerebral oximetry, children, congenital heart disease, near-infrared spectroscopy, near infrared spectroscopy

## Abstract

**Introduction:**

Postoperative acute kidney injury (AKI) is a common postoperative complication in cardiac surgery, with varying reported incidences and prognostic factors. Renal hypoperfusion is believed to be a key factor contributing to postoperative AKI. Near-infrared spectroscopy (NIRS) monitoring, which assesses regional tissue saturation (RSO_2_), has been suggested as a tool to predict postoperative AKI. The aim of this systematic review was to examine the prognostic value of perioperative NIRS monitoring in predicting postoperative AKI in pediatric patients.

**Methods and Results:**

After a systematic search in PubMed, EMBASE, and Cochrane library, twenty studies (1517 patients) were included. The inter-rater agreement on study quality was strong, yet a high risk of bias was identified.

**Conclusion:**

The heterogeneity of the results—in part attributable to several potential confounding factors regarding study population, monitoring technique and the definition of AKI—together with the lack of a clear and consistent association between RSO_2_ values and AKI, currently preclude recommending NIRS monitoring as a reliable and valid clinical tool to “predict” AKI in the individual patient.

## Introduction

Postoperative acute kidney injury (AKI) is a common occurrence in pediatric cardiac surgery, with reported incidences ranging from 3.4%^1^ to 86%.^2^ Although various classification systems have been used in the diagnosis of AKI, the Kidney Disease Improving Global Outcomes (KDIGO) classification is now regarded as the standard diagnostic classification tool.^3^ Biomarkers such as Kidney Injury Molecule-1 (KIM-1) and Neutrophil Gelatinase-Associated Lipocalin (NGAL) have shown a higher sensitivity to detect more subtle forms of renal injury,^4^ but are still mostly used in research settings.^5^

Occurrence of postoperative AKI is dependent on several pre-existing factors, such as young age, chronic kidney disease, anemia, cardiopulmonary bypass duration, multiple cross clamps, and preoperative inotropic support.^6-8^ Perioperative renal hypoperfusion and hypoxia due to intraoperative hypotension^9,10^ have been associated with postoperative AKI.^11^ It has been shown that children developing AKI are more likely to have prolonged mechanical ventilation,^12,13^ longer intensive care unit stay,^14^ and can evolve to chronic kidney disease. The relevance of AKI as a postoperative complication goes beyond (acute or chronic) kidney injury itself, since AKI has also been linked to an increase in morbidity and mortality, with mortality rates up to 89%.^15,16^

The use of near-infrared spectroscopy (NIRS) monitoring, which measures regional tissue saturation (RSO_2_),^17^ enables the real-time detection of local tissue hypoxia. Some authors suggest that renal region tissue oxygenation (RrSO_2_) can be directly monitored by placing NIRS sensors on the flank skin overlying the kidney.^18^

Additionally, assessment of cerebral (RcSO_2_) or somatic (RsSO_2_) tissue oxygenation can be a surrogate for evaluation of global systemic oxygenation and is considered a useful tool in predicting postoperative AKI.^19-22^

Over the last decade, various studies tested the hypothesis that low intraoperative RrSO_2_ values were associated with postoperative AKI, reporting variable findings—and in some centers, RrSO_2_ measurements are routinely performed in cardiac surgery.^19,20,23,24^ The aim of this systematic review was to investigate the prognostic value of perioperative RSO_2_ values to predict postoperative AKI in pediatric patients undergoing cardiac surgery.

## Methods

This systematic review was registered publicly in the International Prospective Register of Systematic Reviews (PROSPERO; registration number: CRD42021241770) and is reported according to the 2020 guidelines from the Preferred Reporting Items for Systematic Reviews and Meta-Analyses (PRISMA) statement.^25^ In this review we choose to focus on the pediatric cardiac surgery population.

### Search Strategy and Article Selection

Three databases were selected for the systematic search (PubMed, EMBASE, and Cochrane Library; supplemental file 1). The database search was conducted on the 18^th^ of February 2021 and repeated on the 18^th^ of February 2024. Since most clinically used AKI definitions have been formulated after 2006,^7,26^ we have included studies from years 2006–2024.^27-29^ We also reviewed the bibliographies of the final selected studies, to identify any other potentially relevant study that was not found through the systematic search.

Studies were considered eligible for inclusion if the following a priori established criteria were met:

1. Original clinical studies, performed in the perioperative period in pediatric patients undergoing cardiac surgery (below 18 years of age).
2. Studies investigating either renal region (RrSO_2_), cerebral (RcSO_2_), or somatic (RsSO_2_) NIRS monitoring in the perioperative phase, defined as the day before surgery (preoperative), during surgery (intraoperative) and the days after surgery (post-operative).
3. Studies investigating the association of any of the RSO_2_ values with postoperative AKI as a primary outcome.
4. Studies using any of the following AKI definitions:
  - KDIGO criteria^3^ or modified KDIGO criteria (based only on serum creatinine, sCr)^30,31^
  - pRIFLE (pediatric Risk, Injury, Failure, Loss, End stage renal disease) criteria^32^
  - AKIN (Acute Kidney Injury Network) criteria^33^
  - (fractional) sCr increase^29^
  - Urine output and/or urinary and serum biomarkers as markers for renal dysfunction (e.g., KIM-1, NGAL, cystatin C, blood urea nitrogen and tissue inhibitor of metalloproteinases-2* insulin-like growth factor-binding protein 7 ([TIMP-2]*[IGFBP7]))^34^
  - estimated glomerular filtration rate

We excluded studies written in a language other than English, animal studies, case reports, review articles and conference abstracts. The search and bibliographic review were performed independently by two researchers (CKN and DM) using the web application Rayyan.^35^

### Definitions of RSO_2_ Values

RSO_2_ values are measured as percentage of tissue hemoglobin oxygen saturation and were presented as absolute values (%; with either mean ± standard deviation or median [interquartile range]), as decrease from baseline value (%), or as predefined calculations. The following RSO_2_ values were presented in the included studies:

• RrSO_2_
  ◦ Absolute RrSO_2_ values (%),^1,30,36-44^ or nadir of absolute RrSO_2_ value (%).^45^
  ◦ Predefined threshold RrSO_2_ values (%) regarding total time (≥2 hours or <2 hours)^12^ of RrSO_2_ below 50%,^2^ or ≥20% reduction from baseline^40^ for at least 60 consecutive seconds,^31^ or 20 minutes.^14^
  ◦ RrSO_2_25 score and RrSO_2_65 score (%min): difference between the baseline RrSO_2_ and the actual RrSO_2_ value (if decreased ≥25% from baseline or if <65%, respectively), multiplied by the time (minutes) during which RrSO_2_ is decreased ≥25% from baseline or is <65%, respectively.^13^
  ◦ RrSO_2_ (%), calculated as a percentage of baseline pulse oximetry saturation (SpO_2_).^46^
• RcSO_2_
  ◦ Absolute RcSO_2_ values,^30,42-44,47^ average RcSO_2_ values (%) at specific time points.^48-50^
  ◦ Predefined threshold RcSO_2_ values (%).^51^
  ◦ RcSO_2_ variability, calculated as the root mean square of successive differences (RMSSD).^23^
• RsSO_2_
  ◦ Absolute RsSO_2_ values,^42^ or average RsSO_2_ value (%) at specific time points.^50^

### Data Extraction and Analysis

The primary outcome variables were the RSO_2_ values and their association with postoperative AKI. The following data were extracted from each study independently: 1. Publication-related data (first author, year, and journal), 2. Number of patients included, 3. Surgery type, 4. NIRS device type and location of sensor(s) placement, 5. RSO_2_ values, 6. AKI diagnostic criteria, 7. AKI incidence (percentage), and 8. Relation of the RSO_2_ values with postoperative AKI.

Data extracted from the studies were tabulated using Microsoft Excel 2010 (Redmond, USA). No meta-analysis was performed, due to the heterogeneity of the included studies. The risk of bias for each article was evaluated independently, using the “Quality Assessment Tool for Observational Cohort and Cross-Sectional Studies” of the National Heart, Lung, and Blood Institute,^52^ after which a consensus on the risk of bias was reached. We used R version 4 (R Core Team, Vienna, Austria, 2020) to determine the inter-rater agreement (Table 1).

**Table 1.** Risk of Bias Check and Overall Rating of the Included Articles as Determined After Reaching Agreement Between the Researchers.

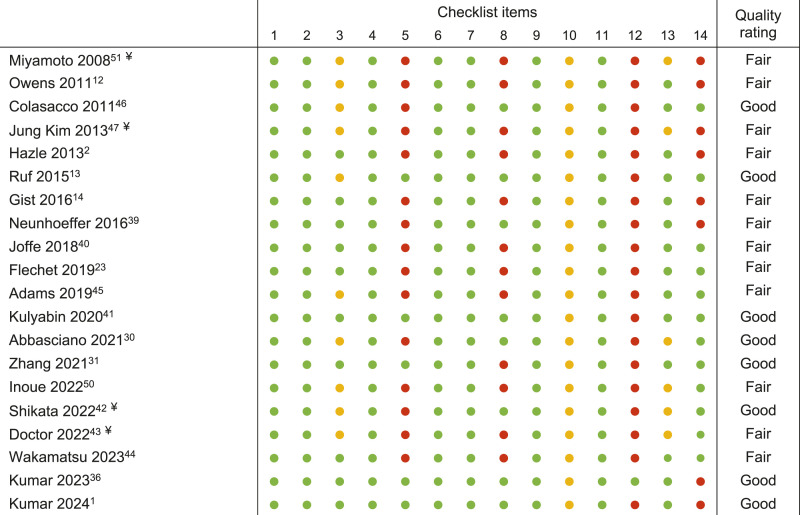

^¥^Retrospective studies; Green = yes, red = no, yellow = not applicable/not reported/cannot determine. Summary of checklist items (the complete assessment tool is available at the following link: https://www.nhlbi.nih.gov/health-topics/study-quality-assessment-tools)^52^: 1. Research question and objective, 2-4. Definition of study population, participation rate, inclusion and exclusion criteria, 5. Sample size definition, 6-8. Relationship between exposure(s) and outcome(s), 9-11. Definition and assessment of exposure(s) and outcome(s), 12. Blinding, 13. Loss to follow-up, 14. Statistical adjustment for confounding variables.

## Results

**Figure 1.**
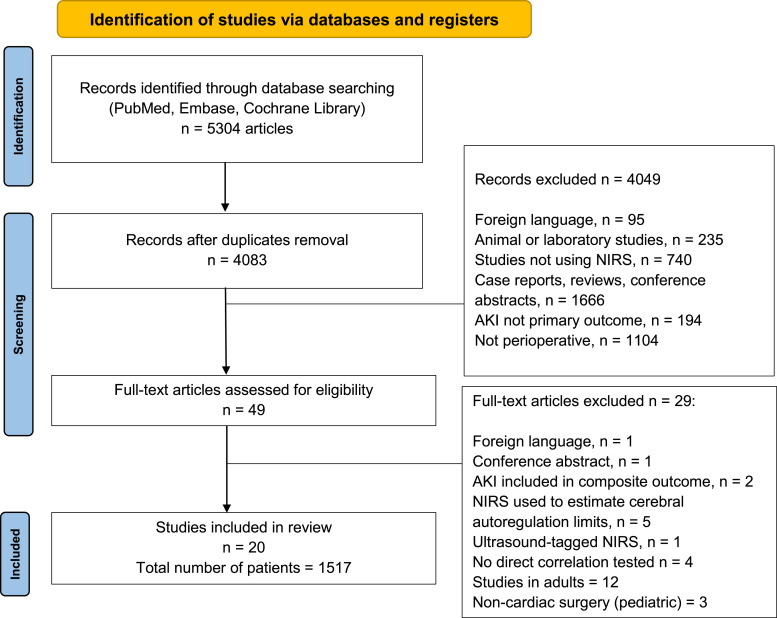
Flowchart with the inclusion and exclusion of articles in this systematic review.

The database search retrieved 5304 studies, of which 1221 were duplicates and were removed. We excluded 4049 studies after reviewing title and abstract, and 49 studies were eligible for full text reviewing. Twenty-nine studies were excluded after full text reviewing and 20 studies were included, comprising 1517 patients with a median [interquartile range] of 57 [47–95] patients per study (Figure 1).

The inter-rater agreement regarding the quality of the studies was “strong”^53^ (percent agreement 86.8%, Cohen’s Kappa = 0.72, 95% CI 0.64–0.80, *P* < .001; Table 1).

A high risk of bias was identified in four main categories: sample size justification, assessment of different levels of exposure, blinding, and statistical adjustment for potential confounding variables.

Most studies included pediatric patients under 12 months of age. Four studies were performed in pediatric patients with non-cyanotic heart disease undergoing aortic arch reconstruction.^41,42,47,51^ Fourteen studies had a mixed population, including both cyanotic and non-cyanotic pediatric patients, undergoing either corrective or palliative procedures.^1,2,12-14,23,30,36,39,40,44-46,50^ The reported incidence of postoperative AKI varied from 3.4%^1^ and 86%.^2^ The diagnostic AKI criteria used were the pRIFLE criteria (n = 7),^12-14,39,43,47,50^ the KDIGO criteria (n = 7),^2,23,40-42,44,45^ a modified KDIGO (using only sCr increase (n = 2),^30,31^ initiation of renal replacement therapy (n = 2),^1,36^ (fractional) sCr increase (n = 2),^12,46^ and urine output (n = 1).^51^

### Renal Region NIRS (RrSO_2_)

Ten out of seventeen studies investigating RrSO_2_ (Table 2) reported that RrSO_2_ values were associated with postoperative AKI.^12,13,30,31,36,39,40,44-46^ The majority of them included both cyanotic and non-cyanotic pediatric patients.

**Table 2.** Overview of the Included Studies.

First Author	Year	Number of Patients	Age	Device	Site of NIRS Monitoring	Period of Monitoring	AKI Definition	AKI Incidence	Comparison Variable	Strength of the Association NIRS-AKI	Other Findings

Miyamoto^51^ ^a^ ^b^	2008	12	0–2 months	nr	Cerebral	Intraoperative	Urine output	nr	RcSO_2_	No association	Higher RcSO_2_ was associated with higher urine output during CPB
Owens^12^	2011	40	0–12 months	INVOS	Renal	Postoperative (48 h)	pRIFLE or sCr increase	25%	RrSO_2_ (<50% for >2 h)	Subjects with RrSO_2_ <50% for >2 h had higher AKI incidence (63% vs 16%, *P* = .015)	
					Cerebral				RcSO_2_		
Colasacco^46^ ^c^	2011	48	0–6 months	nr	Renal	Intraoperative and postoperative (48 h)	Fractional sCr increase	23%	RrSO_2_ (% of baseline SpO_2_)	Correlation RrSO_2_ on POD1 – 40% fractional sCr increase: *R*^2^ = 0.60	
										RrSO_2_ <80% (Sn 100%, Sp 75%) or <75% (Sn 82%, Sp 97%) on POD1	
Jung Kim^47^ ^a^ ^b^	2013	47	0–12 months	INVOS	Renal	Intraoperative	pRIFLE	40.4%	RrSO_2_	No association	
					Cerebral				RcSO_2_		
Hazle^2^	2013	49	0–6 months	INVOS	Renal	Postoperative (24 h)	KDIGO	86%	RrSO_2_ (<50%)	No association	Low RrSO_2_ was associated with elevated urinary NGAL, IL-18, cystatin C and a poor outcome
Ruf^13^ ^c^	2015	59	0–12 months	INVOS	Renal	Intraoperative and postoperative (24 h)	pRIFLE	48%	RrSO_2_25 score	RrSO_2_25 score >23 min%: Sn 67%, Sp 76%, AUROC 0.69	RrSO_2_ performed better than cystatin C and NGAL in predicting AKI
									RrSO_2_65 score	RrSO_2_65 score <95 min%: Sn 96%, Sp 40%, AUROC 0.62	
Gist^14^	2016	106	0–4 years	INVOS	Renal	Intraoperative and postoperative (24 h after CPB)	pRIFLE	32%	RrSO_2_ (decrease ≥20% for 20 min)	No association	Low RrSO_2_ was associated with longer mechanical ventilation and LOS
Neunhoeffer^39^	2016	50	0–12 months	O2C	Renal	Postoperative (at 24 h)	pRIFLE	40%	RrSO_2_	RrSO_2_ <65%: Sn 80%, Sp 65.3%, AUROC 0.75 (repair of CHD)	
										RrSO_2_ <61%: Sn 77.8%, Sp 62.5%, AUROC 0.73 (palliation of CHD)	
Joffe^40^ ^c^	2018	66	Median (IQR): 5.9 (4.6–11.5) months	INVOS	Renal	Intraoperative	KDIGO	65%	RrSO_2_	Baseline RrSO_2_ in the highest tertile: AKI 7.14 times more likely compared to lowest tertile	
										Higher average RrSO_2_ during CPB is associated with increased AKI (OR 1.06)	
Flechet^23^ ^c^	2019	156	0–12 years	Foresight	Cerebral	Postoperative	KDIGO	35%	RcSO_2_ (mean; RMSSD)	Low RcSO_2_ RMSSD associated with higher AKI risk (AUROC 0.68)	A clinical model had better predictive performance than RcSO_2_
Adams^45^ ^c^	2019	70	0–12 months	INVOS	Renal	Intraoperative and postoperative (48 h)	KDIGO	61%	RrSO_2_ (average and lowest value)	Stage 2-3 AKI patients had lower mean RrSO_2_ (66% vs 79% for stage 1 AKI and 84% for no AKI, *P* = .038)	Low RrSO_2_ was associated with elevated NGAL
Kulyabin^41^ ^a^ ^c^	2020	45	0–1 month	nr	Renal	Intraoperative and postoperative (24 h)	KDIGO	42%	RrSO_2_	No association	
					Cerebral						
Abbasciano^30^ ^c^	2021	34	0–6 months	EQUANOX	Renal	Intraoperative and postoperative (min. 24 h)	Modified KDIGO	20%	RcSO_2_	Lower RrSO_2_ values 6 h after surgery were associated with AKI (*P* = .043)	
					Cerebral				RrSO_2_		
Zhang^31^ ^c^ ^d^	2021	242	1–12 months	Foresight	Renal	Intraoperative	Modified KDIGO	31%	RrSO_2_	RrSO_2_ desaturation ≥20% for ≥60 sec is associated with AKI (OR 2.79 (1.21–6.44) *P* = .016)	
Inoue^50^ ^c^ ^d^	2022	87	0–7 months	INVOS	Cerebral	Intraoperative	pRIFLE	23%	RcSO_2_	RsSO_2_ ≤74% is associated with AKI (AUC 0.82, *P* < .001)	
					Abdomen				RsSO_2_		
					Thigh						
Shikata^42^ ^a^ ^b^ ^c^	2022	28	0–3 months	INVOS	Cerebral	Intraoperative	KDIGO	39.3%	RcSO_2_	RsSO_2_ ≤48% for >20 min is associated with KDIGO stage >1 (ROC 0.78, Sn 0.7, Sp 0.78)	
					Renal (flank)				RrSO_2_		
					Thigh				RsSO_2_		
Doctor^43^ ^a^ ^b^	2022	91	0–6 months	INVOS	Cerebral	Postoperative	pRIFLE	33%	RcSO_2_	No association	
					Renal				RrSO_2_		
Wakamatsu^44^	2023	114	0–17 years	INVOS	Cerebral	Intraoperative	KDIGO	35.1%	RcSO_2_	Lower baseline RcSO_2_ were lower in AKI group (61 vs 69.5%; *P* = .031)	
					Renal				RrSO_2_	Lower RcSO_2_ and RrSO_2_ during CPB in AKI group (61 vs 73%; *P* = .013 and 60 vs 77%; *P* = .001). Lower RcSO_2_ and RrSO_2_ nadir in AKI group (48.5 vs 54%; *P* = .039 and 53 vs 71%; *P* = .005). Higher intraoperative RrSO_2_ decrease in AKI group (27.5 vs 13.4%; *P* = .012)	
Kumar^36^	2023	55	0–18 years	INVOS	Cerebral	Intraoperative and postoperative (48 h)	AKI requiring RRT	7.3%	RrSO_2_	Lower RrSO_2_ in AKI patients	
					Renal						
Kumar^1^	2024	118	0–14 years	INVOS	Renal	Intraoperative and postoperative (24 h)	AKI requiring RRT	3.4%	RrSO_2_	No association	No association with NGAL

Abbreviations: pRIFLE, pediatric risk, injury, failure, loss, end stage renal disease; KDIGO, kidney disease: improving global outcomes; modified KDIGO = only use of serum creatinine of the kidney disease: improving global outcomes; sCr, serum creatinine; RrSO_2_, renal tissue oxygenation; RcSO_2_, cerebral tissue oxygenation; RsSO_2_, somatic tissue oxygenation (e.g., muscle); SpO_2_, pulse oximetry saturation, LOS, length of stay; NGAL, neutrophil gelatinase-associated lipocalin; CPB, cardiopulmonary bypass; POD1, postoperative day 1; CHD, congenital heart disease; nr, not reported; RRT, renal replacement therapy.

*R*^2^, coefficient of determination; OR, odds ratio; Sn, sensitivity; Sp, specificity; AUROC, area under the ROC curve; RMSSD, root mean square of successive differences; INVOS (Medtronic, Minneapolis, MN, USA); Foresight (Edwards Lifesciences Corp. Irvine, CA, USA); O2C (LEA Medizintechnik, Giessen, Germany).

^a^Studies including only cyanotic patients.

^b^Retrospective studies.

^c^Relationship between NIRS variables and AKI were risk adjusted.

^d^Studies including only non-cyanotic patients.

The RrSO_2_ was lower in preoperatively cyanotic patients at baseline^12,40^ but not during cardiopulmonary bypass (CPB)^13,40^ and after surgery.^13^ RrSO_2_ values associated with a higher incidence of postoperative AKI were a low perioperative mean RrSO_2_^36,44^ (66% vs 84% in patients without AKI),^45^ intraoperative RrSO_2_ desaturation ≥20% for ≥60 seconds,^31^ an intraoperative RrSO_2_25 score >23 min%^13^ or an intraoperative RrSO_2_65 score <95 min%,^13^ lower RrSO_2_ values at 6 hours postoperatively,^30^ a postoperative RrSO_2_ <50% for >2 hours,^12^ a postoperative mean RrSO_2_ <80% (higher sensitivity) or <75% (higher specificity) of individual baseline SpO_2_,^46^ and a postoperative RrSO_2_ <65% or <61% (corrective or palliative surgical procedures, respectively).^39^ The lowest intraoperative RrSO_2_ value predicted postoperative increases in urinary NGAL and [TIMP-2]*[IGFBP7],^45^ and patients spending more cumulative time with postoperative RrSO_2_ values <50% had increased postoperative levels of urinary biomarkers (NGAL, IL-18 and cystatin C).^2^ In one study higher RrSO_2_ values before or during CPB were associated with the development of postoperative AKI.^40^ The remaining seven studies (of which four including solely cyanotic patients^41-43,47^) did not find an association between RrSO_2_ and postoperative AKI.^1,13,14,41-43,47^

### Cerebral NIRS (RcSO_2_)

Pediatric patients who developed AKI had a lower maximum RcSO_2_ value (76% vs 79%) and a lower RMSSD (variability of RcSO_2_ that takes gradual shifts in mean into account) as reported by one study.^23^ Another study reported lower RcSO_2_ values before surgery (61% vs 69.5%) and during CPB (61% vs 73%) in patients developing AKI.^44^ The other eight studies found no association between RcSO_2_ values and postoperative AKI in cyanotic patients,^42,43,47,51^ in a mixed cyanotic and non-cyanotic population,^12,30^ and in non-cyanotic patients.^31,50^

### Somatic NIRS (RsSO_2_)

RsSO_2_ values ≤74% measured at the thigh muscle in non-cyanotic patients undergoing ventricular septum defect repair were associated with postoperative AKI.^50^ In aortic arch reconstruction with high-flow regional cerebral perfusion, a RsSO_2_ ≤48% for >20 min was associated with development of postoperative AKI (KDIGO stage >1).^42^

## Discussion

The aim of this systematic review was to investigate the prognostic value of perioperative RSO_2_ values to predict postoperative AKI in pediatric patients undergoing cardiac surgery. The findings of this review preclude the use of NIRS monitoring as a valid clinical tool to predict postoperative AKI.

### Key Findings

Low RrSO_2_ values were associated with postoperative AKI in nine out of seventeen studies. One of these seventeen studies reported an association between higher intraoperative RrSO_2_ values and postoperative AKI. Only two out of nine studies reported an association of postoperative AKI with low RcSO_2_ values in pediatric cardiac surgery. Postoperative AKI was associated with lower RsSO_2_ in both studies reporting this measurement. Threshold RSO_2_ values under which postoperative AKI was likely to develop were extremely variable, making it difficult to establish threshold values for clinical practice. Therefore, the current evidence precludes recommending the use of NIRS monitoring as a valid clinical tool to predict AKI in the individual patient.

### Study Population, Monitoring Site, and AKI Definition

A challenge to drawing comprehensive conclusions stemmed from the inclusion of patients with both cyanotic and non-cyanotic heart disease, which represent distinct pathophysiological profiles with varying baseline RrSO_2_ values.^40^ In studies involving a mixed patient population, low RrSO_2_ and/or RcSO_2_ values were associated with AKI in eight and two out of 13 studies, respectively. None of the studies involving cyanotic pediatric patients found an association between RrSO_2_, RcSO_2_, and the development of postoperative AKI. In cyanotic pediatric patients,^40^ low preoperative arterial oxygen saturation could play a protective role against perioperative kidney dysfunction, by a chronic reduction of metabolic rate of the brain and kidneys (“ischemic preconditioning”)^54^ keeping RrSO_2_ and RcSO_2_ values close to their preoperative baseline values.

RcSO_2_ values have been suggested to be a surrogate of tissue oxygenation in general^55^ and could be an indirect RrSO_2_ monitor.^24,56^ The prognostic value of RcSO_2_ to predict AKI in the pediatric population seems lower compared with RrSO_2_ values or RsSO_2_ values. A possible explanation is that the kidneys and other tissues (like muscles) suffer from hypoperfusion before the brain, due to hierarchical distribution of blood flow^57^ and differences in organ blood flow autoregulation.^58^

Proper placement of NIRS sensors for measuring RrSO_2_ may contribute to the variability of the results. Only three studies reported the use of ultrasound imaging when placing the NIRS sensor above the renal region,^14,31,39^ instead of relying on external anatomy landmarks. Two of these studies found an association between low RrSO_2_ values and AKI, emphasizing the contribution of contamination of the NIRS signal by extrarenal tissues in acquiring reliable RrSO_2_ measurements.^18^

In addition, the depth of the kidney beneath skin surface is dependent on the patient’s size and weight, and the average depth of penetration of NIRS sensors is generally around 2 cm in children. It is possible that if the kidney is located “too deep” (e.g., in overweight patients or older infants), the NIRS device may not be accurately measuring renal tissue oxygenation. Even if the renal cortex is reached by the near-infrared light, the renal medulla—physiologically more sensitive to hypoxia than the cortex^59^—might remain out of the reach of renal region NIRS monitoring.

Diagnostic criteria used for diagnosing postoperative AKI were highly variable, reflected by the wide range of reported AKI incidences. It is possible that due to these differing criteria, the association between NIRS values and postoperative AKI may have been obscured. In pediatric patients we found some evidence for an association between RrSO_2_ and novel biomarkers of kidney injury—mainly NGAL and cystatin C—suggesting that RrSO_2_ might detect subtle alterations in renal function which may go undetected using conventional AKI criteria.

### Strengths and Limitations

To the authors knowledge, this is the first systematic review about the use of perioperative RSO_2_ to predict postoperative AKI in pediatric cardiac surgery. A certified information specialist helped develop a complete search strategy, which would unlikely develop selection bias. We performed a validated quality assessment of the selected studies and determined an inter-rater variability to objectify the differences in opinion. The heterogeneity of the included studies (regarding, for example, methodological differences in NIRS devices and the reporting of NIRS values, and the different AKI definitions used), prevented us from performing a meta-analysis, making this a descriptive review with a systematic search.

### Advice for Future Research

In pediatric cardiac surgery, future studies on this topic should classify patients based on cardiac pathophysiology (cyanotic vs non-cyanotic).^60^ The focus should be on the monitoring of RrSO_2_ and RsSO_2_ values, since they might predict postoperative AKI more consistently than RcSO_2_. The proper positioning of NIRS sensors in the renal region should be guided by ultrasound imaging. The definition of RSO_2_ thresholds could be based on deviations from individual RSO_2_ baseline values rather than on absolute values, which might be less interpretable due to a wide variability in the population. The KDIGO criteria are currently advised in clinical practice^34^ and should therefore be applied to detect AKI in future studies. We do not expect that the standardization of the population, monitoring and outcome in other observational studies will allow the definition of a single regional oxygenation threshold capable of predicting AKI, because of the multiple factors interplaying in determining kidney injury. It is likely that recommendations for threshold RSO_2_ values will continue to rely primarily on consensus and expert opinions, rather than on consistent data from observational studies. We would suggest designing interventional studies to determine if a “NIRS-directed” respiratory and hemodynamic perioperative management could potentially reduce the incidence of postoperative AKI.

## Conclusion

Across twenty studies conducted in pediatric patients undergoing cardiac surgery, no clear or consistent association was found between RSO_2_ values and postoperative AKI. This currently precludes recommending the use of perioperative near-infrared spectroscopy monitoring as a reliable clinical tool for predicting postoperative acute kidney injury in this population.

## Supplemental Material

Supplemental Material - Prognostic Value of Perioperative Near-Infrared Spectroscopy Monitoring for Postoperative Acute Kidney Injury in Pediatric Cardiac Surgery: A Systematic Review Supplemental Material for Prognostic Value of Perioperative Near-Infrared Spectroscopy Monitoring for Postoperative Acute Kidney Injury in Pediatric Cardiac Surgery: A Systematic Review by Cornelia K. Niezen, Marco Modestini, Dario Massari, Arend F. Bos, Thomas W. L. Scheeren, Michel M. R. F. Struys, and Jaap Jan Vos in Seminars in Cardiothoracic and Vascular Anesthesia

## Appendix

### Glossary of Terms

AKI: Acute kidney injury
AKIN: Acute kidney injury network
KDIGO: Kidney disease improving global outcomes
KIM-1: Kidney injury molecule-1
NGAL: Neutrophil gelatinase-associated lipocalin
NIRS: Near-infrared spectroscopy
pRIFLE: Pediatric risk, injury, failure, loss, end stage renal disease
PRISMA: Preferred reporting items for systematic reviews and meta-analyses
PROSPERO: International prospective register of systematic reviews
RcSO_2_: Cerebral tissue oxygenation
RIFLE: Risk, injury, failure, loss, end stage renal disease
RMSSD: Root mean square of successive differences
RSO_2_: Regional tissue saturation
RrSO_2_: Renal region tissue oxygenation
RsSO_2_: Somatic tissue oxygenation
sCr: Serum creatinine
[TIMP-2]*[IGFBP7]: Tissue inhibitor of metalloproteinases-2* insulin-like growth factor-binding protein 7.
