# Supplementary material for: Prognostic Value of Perioperative Near-Infrared Spectroscopy Monitoring for Postoperative Acute Kidney Injury in Pediatric Cardiac Surgery: A Systematic Review
Source: Semin Cardiothorac Vasc Anesth. 2025 Feb 10;29(3):209–18. doi: 10.1177/10892532251316682 (PMC12340143; doi:10.1177/10892532251316682)
Supplement: Supplemental Material - Prognostic Value of Perioperative Near-Infrared Spectroscopy Monitoring for Postoperative Acute Kidney Injury in Pediatric Cardiac Surgery: A Systematic Review [file sj-pdf-1-scv-10.1177_10892532251316682.pdf]

Supplementary file 1: Search strategy used for Pubmed, Embase and Cochrane Library

Pubmed:

("Spectroscopy, Near-Infrared"[Mesh] OR Near-infrared spectroscop*[tiab] OR near-infra red spectroscop*[tiab] OR NIRS[tiab] OR tissue oxygenation[tiab] OR StO2[tiab] OR rSO2 [tiab] OR rcSO2[tiab] OR cerebral oximetr*[tiab] OR nir spectroscop*[tiab] OR inspectra[tiab] OR invos[tiab] OR foresight[tiab] OR portamon[tiab] OR oxiplex[tiab] OR niro[tiab] OR Masimo[tiab] OR oxygen saturation*[tiab])

AND

("Acute Kidney Injury"[Mesh] OR "Renal Insufficiency"[Mesh] OR AKI[tiab] OR kidney injur*[tiab] OR renal injur*[tiab] OR ARF[tiab] OR acute renal failur*[tiab] OR renal insufficienc*[tiab] OR KDIGO[tiab] OR RIFLE[tiab] OR pRIFLE[tiab] OR AKIN[tiab] OR creatinin*[tiab] OR urine output[tiab] OR urin*[tiab] OR biomarker*[tiab] OR kidney damag*[tiab] OR NGAL[tiab] OR kidney function[tiab] OR renal insult[tiab] OR BUN[tiab] OR oliguria[tiab] OR anuria[tiab] OR polyuria[tiab] OR "Renal Dialysis"[Mesh] OR glomerular[tiab] OR CRRT[tiab] OR renal replacement[tiab] OR ATN[tiab])

AND

("Thoracic Surgery"[Mesh] OR "Cardiovascular Surgical Procedures"[Mesh] OR "Heart Defects, Congenital"[Mesh] OR "Cardiopulmonary Bypass"[Mesh] OR cardiac surger*[tiab] OR congenital heart diseas*[tiab] OR cyanot*[tiab] OR shunt[tiab] OR CHD[tiab] OR cardiovascular procedur*[tiab] OR CPB[tiab] OR "Surgical Procedures, Operative"[Mesh] OR "surgical procedures, operative"[MeSH] OR "general surgery"[MeSH] OR surger*[tiab] OR "Perioperative Period"[MeSH] OR

"Perioperative Medicine"[MeSH] OR perioperat*[tiab] OR peri-operat*[tiab] OR intraoperat*[tiab] OR intra-operat*[tiab] OR "Postoperative Complications"[Mesh] OR postoperat*[tiab] OR post-operat*[tiab] OR bypass*[tiab] OR procedure*[tiab] OR cardiac*[tiab] OR “Operating Rooms”[Mesh] OR operat*[tiab])

Embase:

('near infrared spectroscopy'/exp OR 'Near-infrared spectroscop*':ab,ti,kw OR 'near-infra red spectroscop*':ab,ti,kw OR 'NIRS':ab,ti,kw OR 'tissue oxygenation':ab,ti,kw OR 'StO2':ab,ti,kw OR 'rSO2':ab,ti,kw OR 'rcSO2':ab,ti,kw OR 'cerebral oximetr*':ab,ti,kw OR 'nir spectroscop*':ab,ti,kw OR 'inspectra':ab,ti,kw OR 'invos':ab,ti,kw OR 'foresight':ab,ti,kw OR 'portamon':ab,ti,kw OR 'oxiplex':ab,ti,kw OR 'niro':ab,ti,kw OR 'Masimo':ab,ti,kw OR 'oxygen saturation*':ab,ti,kw)

AND

('AKI':ab,ti,kw OR 'kidney injur*':ab,ti,kw OR 'renal injur*':ab,ti,kw OR 'ARF':ab,ti,kw OR 'acute renal failur*':ab,ti,kw OR 'renal insufficienc*':ab,ti,kw OR 'KDIGO':ab,ti,kw OR 'RIFLE':ab,ti,kw OR 'pRIFLE':ab,ti,kw OR 'AKIN':ab,ti,kw OR 'creatinin*':ab,ti,kw OR 'urine output':ab,ti,kw OR 'urin*':ab,ti,kw OR 'biomarker*':ab,ti,kw OR 'kidney damag*':ab,ti,kw OR 'NGAL':ab,ti,kw OR 'kidney function':ab,ti,kw OR 'renal insult':ab,ti,kw OR 'BUN':ab,ti,kw OR 'oliguria':ab,ti,kw OR 'anuria':ab,ti,kw OR 'polyuria':ab,ti,kw OR 'glomerular':ab,ti,kw OR 'CRRT':ab,ti,kw OR 'renal replacement':ab,ti,kw OR 'ATN':ab,ti,kw OR 'Risk, Injury, Failure, Loss of kidney function and End-stage kidney disease classification'/exp OR 'kidney failure'/exp OR 'kidney injury'/exp OR 'dialysis'/exp OR 'continuous renal replacement therapy'/exp)

AND

('cardiac surger*':ab,ti,kw OR 'congenital heart diseas*':ab,ti,kw OR 'cyanot*':ab,ti,kw OR 'shunt':ab,ti,kw OR 'CHD':ab,ti,kw OR 'cardiovascular procedur*':ab,ti,kw OR 'CPB':ab,ti,kw OR 'surger*':ab,ti,kw OR 'perioperat*':ab,ti,kw OR 'peri-operat*':ab,ti,kw OR 'intraoperat*':ab,ti,kw OR 'intra-operat*':ab,ti,kw OR 'postoperat*':ab,ti,kw OR 'post-operat*':ab,ti,kw OR 'bypass*':ab,ti,kw OR 'procedure*':ab,ti,kw OR 'cardiac*':ab,ti,kw OR 'operat*':ab,ti,kw OR 'surgery'/exp OR 'perioperative monitoring'/exp OR 'Perioperative Period'/exp OR 'Perioperative Medicine'/exp OR 'Postoperative Complications'/exp OR 'operating room'/exp OR 'heart surgery'/exp OR 'cardiac surgery intensive care unit'/exp OR 'congenital heart disease'/exp OR 'congenital heart malformation'/exp)

Cochrane search:

1.“near-infrared spectroscopy” OR “near infrared spectroscopy” OR “NIRS” OR Nir spectroscopy” OR “tissue oxygenation” OR “regional tissue oxygenation” OR “StO2” OR “rSO2” OR “rcSO2” OR “oxygen saturation” OR “cerebral oximetry” OR “Inspectra” OR “INVOS” OR “foresight” OR “portamon” OR “oxiplex” OR “niro” OR “Massimo”

2. "Acute Kidney Injury" OR "Renal Insufficiency" OR “AKI”OR “kidney injury” OR “renal injury” OR “ARF” OR “acute renal failure” OR “renal insufficiency” OR “KDIGO” OR “RIFLE” OR “pRIFLE” OR “AKIN” OR “creatinine” OR “urine output” OR “urine” OR “biomarker” OR “biomarkers” OR “kidney damage” OR “NGAL” OR “kidney function” OR “renal insult” OR “BUN” OR “oliguria” OR “anuria” OR “polyuria” OR "Renal Dialysis" OR “glomerular” OR “CRRT” OR “renal replacement” OR “ATN”

3. "Thoracic Surgery" OR "Cardiovascular Surgical Procedures" OR "Congenital Heart Defects" OR "Cardiopulmonary Bypass" OR "cardiac surgery" OR "congenital

heart disease" OR "cyanotic" OR "shunt" OR "CHD" OR "cardiovascular procedure" OR "CPB" OR "Surgical Procedures" OR "Operative" OR "surgical procedures" OR "general surgery" OR "surgery" OR "Perioperative Period" OR "Perioperative Medicine" OR "perioperative" OR "peri-operative" OR "intraoperative" OR "intra-operative" OR "Postoperative Complications" OR "postoperative" OR "post-operative" OR "bypass" OR "procedure" OR "cardiac" OR "Operating Rooms" OR "operation"
